# Coronavirus Disease 2019 in Neonates – What Is Known and What Needs to Be Known

**DOI:** 10.7759/cureus.10171

**Published:** 2020-08-31

**Authors:** Manas Nayak, Santosh Panda, Janaki Ballav Pradhan, Nirmal K Mohakud

**Affiliations:** 1 Neonatology, Kalinga Institute of Medical Sciences, Bhubaneswar, IND; 2 Pediatric Medicine, Kalinga Institute of Medical Sciences, Bhubaneswar, IND

**Keywords:** covid-19, vertical transmission, newborn, sars cov-2

## Abstract

The severe acute respiratory syndrome coronavirus 2 (SARS-CoV-2) first broke out in Wuhan, China, and subsequently spread worldwide affecting all ages including newborns. The cases of coronavirus disease 2019 (COVID-19) are very less in neonates and they recover well with supportive treatment. Though the vertical transmission of infection is scarcely found, to get rid of acquiring infection by horizontal transmission one should screen all pregnant women and ensure standard infection control measures and monitoring of newborns at risk of COVID-19. Neonates may present as a refusal to feed, feeding intolerance, fever, pneumonia, shortness of breath, and lethargic. Based on available evidence, antivirals like lopinavir, ritonavir, remdesivir specific medication like chloroquine/hydroxychloroquine, azithromycin, corticosteroids, and intravenous gamma globulin, are not recommended, so early detection and supportive treatment is needed for an optimal outcome.

## Introduction and background

The novel coronavirus disease 2019, caused by severe acute respiratory syndrome Coronavirus 2 (SARS-CoV-2), was named coronavirus disease (COVID-19) by the World Health Organization on January 7, 2020 [1]. A small proportion of cases of COVID-19 caused by SARS-CoV-2 infection have been reported in neonates compared to the total number of cases in the general population. As there are approximately 0.35 million deliveries per year as per the United Nations Children's Fund (UNICEF), screening of pregnant women and perinatal care in babies of COVID-19 positive mothers is very important. Initially, there was insufficient evidence of mother-to-infant transmission and only a few deaths were reported in newborns [2,3], but now transplacental transmission has been established in a few cases [4]. In this review, an attempt is made to discuss the clinical features, diagnosis, management, and preventive strategies of neonates born during the COVID-19 outbreak.

## Review

Vertical and horizontal transmission

Pregnancy is an immunosuppressed condition and normal physiologic and immunologic changes that occur during this period increase the risk of respiratory tract infection-related complications. The risk of maternal morbidity and mortality is increased much more in pregnancy in comparison to non-pregnant women during influenza attacks [5]. The same association has been found previously in pregnant women infected with severe acute respiratory syndrome (SARS) and the Middle East respiratory syndrome (MERS) [6]. Prenatal or perinatal transmission of SARS-CoV-2 to newborns during pregnancy is not established. Hence we should understand the antepartum, intrapartum, and neonatal factors that influence perinatal transmission and which maternal body fluids are infectious.

Respiratory secretions and saliva are mainly responsible for human-to-human transmission of SARS-CoV-2. The virus in blood and stool were also detected in non-pregnant women with severe illness [7]. Initial studies in China did not show any virus in vaginal mucus, amniotic fluid, serum, blood, and/or breast milk, but found in a nasopharyngeal swab of the mother [2,3]. Later on, Kirtsman et al. showed congenital SARS-CoV-2 infection where SARS-CoV-2 ribonucleic acid (RNA) was found in a nasopharyngeal swab of the neonate, placental tissue, breast milk, and vaginal swab [8]. The SARS-CoV-2-specific immunoglobulin (Ig)M and IgG were detected in three newborns of infected women, suggesting the possibility of vertical transmission which triggers the production of IgM antibody in the fetus [9,10], but like other diseases, false-positive IgM results limit its interpretation and we need further studies to confirm the possibility of in utero transmission [11].

Clinical presentation

Presently few positive confirmed neonatal COVID-19 cases have been found and all of them had no symptoms or very mild to moderate symptoms [12,13]. One study of neonates delivered from COVID-19 positive mothers had found respiratory distress, gastrointestinal symptoms, fever, tachycardia, and vomiting, but babies were SARS-CoV-2 negative [14]. In another study few babies present with a rash with varied distribution and one baby presented with transient tachypnea of newborns (TTNB) [15]. In a small group of three neonates with COVID-19, symptoms like shortness of breath, fever, lethargy, refusal feed were observed with nonspecific radiographic findings, and no deaths [16].

Diagnosis

As the nature of vertical transmission is not clear and the horizontal transmission risk during the immediate postnatal period is a possible way for transmission to the newborn baby, the timing of testing, the method of testing, and their interpretation remain unclear [8]. The diagnosis of SARS-CoV-2 is done by real-time polymerase chain reaction (RT-PCR), taking samples from upper or lower airway secretions. It is seen that higher viral loads of SARS-CoV-2 are there in samples collected from the lower airways [17]. Therefore, an initial negative result on nasopharyngeal or throat swab should be repeated or samples from the lower respiratory tract should be taken if possible. In some cases, stool samples are found positive for novel coronavirus but till now not used for SARS-CoV-2 diagnosis [18]. Diagnosis of SARS-CoV-2 by using RT-PCR from blood samples had also been reported [19]. Antigen-based rapid diagnostic tests (RTD) for patients with other concurrent viral infections like influenza have a sensitivity of 34% to 80% [20]. Serology for the diagnosis of SARS-CoV-2 is not very useful in the early phase as the usual antibody response develops in the second week after the onset of the disease [21]. In some confirmed cases by RT-PCR, there is a variable outcome of weak, late, or absent antibody responses detected [21,22]. The serology test has shown positivity in some studies, but the interpretation has to taken cautiously as a high chance of false positivity was noted [10]. Again there is cross-reactivity between antibodies of common CoVs and SARS-CoV-2 has been observed [23].

The American Academy of Pediatrics (AAP) recommends RT-PCR to be tested at 24hrs and repeat at 48hrs in neonates who are born to infected and test positive mothers. But as of now, the World Health Organization (WHO) does not recommend the antigen or antibody-detecting rapid diagnostic tests for treatment purposes, due to the inadequacy of data. At the same time, more research on their diagnostic utility should be persuaded

Treatment

Antepartum Care

To date, limited data is available on maternal COVID-19 infection and its impact on the fetus. Viral pneumonia in pregnant women is associated with adverse neonatal outcomes [24]. There is an increased risk of preterm birth, intrauterine growth restriction, low birth weight, and perinatal depression compared with those without pneumonia as per nationwide population-based data [25]. When a pregnant woman is a suspected or positive case of COVID-19, monitoring of fetal growth by ultrasound assessment every two to four weeks is essential [26]. Though there is no evidence of vertical transmission in women with COVID-19 in late pregnancy, fetal infection during the first and early second trimester of pregnancy is yet to be known [2,3].

There is no evidence of harmful effects of antenatal steroids given for lung maturation in COVID-19 positive pregnant women, hence steroids should be given to pregnant women who are having a risk of preterm delivery as suggested by British Association of Perinatal Medicine (BAPM) guideline-2020, but in a critically ill patient, it should be cautiously given after multidisciplinary consultation due to its harmful effect [27]. In the case of a confirmed COVID-19 positive pregnant woman with spontaneous preterm labor, delivery shouldn't be delayed by using tocolysis. Magnesium sulfate (MgSO4) should be given to pregnant women <30 weeks gestation with the risk of preterm delivery for neuroprotection as per the current guidelines.

Intrapartum Care

The clinical condition of the mother, gestational age of the fetus should be the main criteria in deciding the mode and timing of delivery. If the mother is suspected or confirmed COVID-19 at the time of delivery, neonates should be kept under observation and monitor for infection. After delivery, the umbilical cord should be clamped promptly and the neonate should be transferred to the newborn baby corner which is 6 feet distance from the delivery site for care by the pediatric unit. Isolation is needed for suspected/probable cases, but confirmed cases should be managed in a negative-pressure isolation room. At present, there is inadequate data to recommend delayed cord clamping which may increase the chance of neonatal infection [27,28]. Clinicians attending resuscitation should use complete personal protective equipment (PPE) as there is a possibility of aerosol-generating procedures like airway suction, intubation, and positive pressure ventilation. Neonatal resuscitation should be done according to the Neonatal Resuscitation Program (NRP) guidelines [28].

Breastfeeding

Mothers milk is the best nutrition available at the time of birth and helps in enhancing immunity thereby preventing many viral illnesses. Till now with the limited available evidence, it shows that the virus does not pass through breast milk from an infected mother with SARS-CoV-2 [29]. But there are controversial approaches with differences in recommendations from different societies. American Academy of Pediatrics, the Chinese expert consensus and the US Centers for Disease Control and Prevention, recommends isolation of the neonates immediately after delivery, formula or expressed breast milk feeding, and no contact with the mother for 14 days or at least seven days from symptoms onset. At the same time WHO, UK Royal College of Obstetricians & Gynecologists, and the Italian Society of Neonatology recommendations advocate for the promotion of breastfeeding and the initial mother-infant relationship after childbirth with zero separation but taking appropriate hand hygiene practice and using protective gown and mask. A recent study by Salvatore and colleagues did not find any risk of transmission with breastfeeding and rooming in with mother and newborn when the mother is properly masked [30]. A healthy caregiver should feed the expressed breast milk in an isolated room. In case the mother or parent wanted to be room-in and she wishes to feed directly she must wear a face mask and follow the hand hygiene practice for each feeding.

Management of Asymptomatic Neonates

Considering the high risk of viral transmission from symptomatic COVID-19 infected mother (with fever, cough, cold required intensive care), the asymptomatic neonate should be separated from the symptomatic mother immediately after birth and taken care of by a healthy caregiver. The neonate should be fed with express breast milk, pasteurized donor human milk, or formula feeding. All healthy term or late-preterm neonates born to asymptomatic COVID-19 positive mothers are allowed to be rooming in with the mother and encouraged for direct breastfeeding. The neonate should be kept more than two meters away from his or her mother and looked after by a healthy caregiver. The mother should wear a face mask, use alcohol-based hand rub, and practice hand hygiene for the prevention of postnatal transmission. Routine neonatal monitoring like adequacy of breastfeeding, jaundice, thyroid function test, pulse oximetry screening, and routine immunization should be carried out.

Management of Symptomatic Neonates 

All inborn or outborn symptomatic neonates delivered to suspected or proven COVID-19 infected mother, should be admitted in separate isolation neonatal intensive care unit with negative pressure area and taken care by separate doctors, nursing, and support staff with personal protective equipment. All neonatal symptoms are managed similarly to previous disease-specific own unit protocol with special attention to avoid aerosol generation. Airway suctioning, intubation, surfactant administration, nebulization, extubation are known aerosol-generating procedures in the neonatal intensive care unit. To avoid aerosol production during endotracheal intubation, a neonate with respiratory distress should be initiated with non -invasive ventilation and lung-protective strategy during invasive mechanical ventilatory support. Noninvasive positive-pressure ventilation (NIPPV) or nasal continuous positive airway pressure (NCPAP) should be preferred over high flow nasal cannula (HFNC) as primary mode and weaning modality during the post-extubation period. Minimal gas flow is used during nCPAP to produce gentle bubbling for optimal positive end-expiratory pressure [31]. Interventions like the use of the tight nasal interface in non-invasive ventilation, pre-attached nebulization chamber, use of a cuffed endotracheal tube, or appropriate sized endotracheal tube (ET) to avoid ET leaking, high-efficiency particulate air (HEPA) or viral filter in the respiratory circuit, inline suction could prevent aerosol generation.

In a case series of four full-term neonates delivered to COVID-19 positive mothers, one infant had respiratory distress managed by nasal continuous positive airway pressure (nCPAP) and two infants had rashes at birth and one had facial ulcerations [15]. In both series, nasopharynx swab RT-PCR was negative among all neonates. In a case study Zeng et al., three neonates among 33 babies born to COVID-19 positive mothers had pneumonia with positive viral detection by RT-PCR [16]. All neonates should closely monitor for early diagnosis of common neonatal morbidities and COVID-19 symptoms like temperature instability, apnea, respiratory distress, GI symptoms, and hypotension. Apart from SARS-CoV-2 pneumonia, even COVID-19 positive neonates developed respiratory distress secondary TTNB, respiratory distress syndrome, meconium aspiration syndrome, persistent pulmonary hypertension, and air-leak syndrome.

Based on available evidence, antivirals like lopinavir, ritonavir, remdesivir, or specific medication like chloroquine/hydroxychloroquine, azithromycin, systemic corticosteroids, Granulocyte-macrophage colony-stimulating factor (GM-CSF) and intravenous gamma globulin are not recommended in symptomatic or asymptomatic COVID-19 neonates [31-33]. 

## Conclusions

Very few cases of COVID-19 are reported in neonates. As vertical transmission is unlikely, the SARS-CoV-2 positive mother should wear a face mask and practice hand hygiene for the prevention of postnatal transmission.The COVID-19 neonates recovered well with minimal treatment. Antiviral and hydroxychloroquine not recommended for neonates. A safe vaccine is a future hope. Therefore, it is very important to screen all pregnant women and ensure standard infection control measures and monitoring of newborns with risk of COVID-19.
